# Lipoprotein sialylation in atherosclerosis: Lessons from mice

**DOI:** 10.3389/fendo.2022.953165

**Published:** 2022-09-06

**Authors:** Liming Yu, Jun Peng, Chieko Mineo

**Affiliations:** ^1^Center for Pulmonary and Vascular Biology, Department of Pediatrics, University of Texas Southwestern Medical Center, Dallas, TX, United States; ^2^Department of Cell Biology, University of Texas Southwestern Medical Center, Dallas, TX, United States

**Keywords:** atherosclerosis, sialic acid, neuraminidase, sialyltransferase, lipoprotein, selectin

## Abstract

Sialylation is a dynamically regulated modification, which commonly occurs at the terminal of glycan chains in glycoproteins and glycolipids in eukaryotic cells. Sialylation plays a key role in a wide array of biological processes through the regulation of protein–protein interactions, intracellular localization, vesicular trafficking, and signal transduction. A majority of the proteins involved in lipoprotein metabolism and atherogenesis, such as apolipoproteins and lipoprotein receptors, are sialylated in their glycan structures. Earlier studies in humans and in preclinical models found a positive correlation between low sialylation of lipoproteins and atherosclerosis. More recent works using loss- and gain-of-function approaches in mice have revealed molecular and cellular mechanisms by which protein sialylation modulates causally the process of atherosclerosis. The purpose of this concise review is to summarize these findings in mouse models and to provide mechanistic insights into lipoprotein sialylation and atherosclerosis.

## Introduction

Sialic acids are a family of negatively charged carbohydrates, which are commonly found as terminal residues of an oligosaccharide chain of glycoproteins or glycolipids in the eukaryotic cells. The terminal sialylation plays a key role in diverse biological functions, including the regulation of leukocyte–endothelial cells interaction, signal transduction, and maintenance of normal protein conformation and intracellular transport (1–8). Aberrant sialylation has been implicated in several diseases, including cancer, pathogen infection, and cardiovascular disease (CVD) (9–11). Previous reports in large cohorts have found that high serum levels of sialic acid, both protein-bound and free forms, are an independent risk factor for CVD (12, 13). Other studies have shown a positive relationship between plasma total sialic acid levels and severity or complications of CVD (14–16), while two reports have failed to find such associations (17, 18). Many apolipoproteins, including apolipoprotein B (ApoB), apolipoprotein C-III (ApoC-III), and apolipoprotein (ApoE) are highly glycosylated, with sialic acids being the predominant terminal residues (19–23). The addition or removal of sialic acid in apolipoproteins is regulated by a group of enzymes, namely, neuraminidases (also known as sialidases) and sialyltransferases, and an alteration of expression or activity of these enzymes are known to influence lipoprotein metabolism and atherogenesis (24–26). Increased neuraminidase activity has been described in the plasma of patients after myocardial infarction compared with that in healthy controls (27). Upregulation of sialyltranferase activity has also been shown in atheroma and in the plasma from individuals with atherosclerosis compared to heathy donors (28). However, it is entirely unknown whether these changes in the enzyme activities impact circulating levels of sialic acids or sialylation of proteins related to the atherogenesis in humans.

ApoB is the main structural component of very-low-density lipoprotein (VLDL) and low-density lipoprotein (LDL), and it contains a number of glycosylation sites, majority of which are modified with complex N-linked glycans with sialic acid as terminal residue (29). Earlier works demonstrated that patients with CVD or type 2 diabetes (T2DM) have elevated levels of plasma LDL containing lower amounts of sialic acids compared to healthy individuals (19, 30). These hyposialylated LDL particles have been characterized as smaller in size with an increased electronegative charge, containing more triglyceride (TG), fatty acid, and oxysterols compared to normally sialylated LDL (31–34). Mechanistically, desialylated-LDL particles generated by neuraminidase treatment *ex vivo* were more readily taken up by cells isolated from human aortas than native LDL, leading to increased intracellular accumulation of cholesterol ester (25, 35, 36). Both conventional scavenger receptors and galactose-specific lectin receptors such as asialoglycoprotein receptors (ASGRs), which recognize terminal galactose residues that are exposed after desialylation, have been implicated to facilitate uptake of desialylated LDL (25, 35, 36). Desialylated LDL is also shown to be highly immunogenic, and it can induce the production of proatherogenic autoantibodies (32, 37–39). More recent studies in cultured cells found that neuraminidase-mediated desialylation of high-density lipoprotein (HDL) particles impairs their ability to mediate reverse cholesterol transport (RCT) (35, 40).

ApoC-III is another apolipoprotein in which a relationship between its sialylation status and capacity to regulate lipid metabolism is well established. ApoC-III is mainly synthesized in the liver, and in the circulation, it is carried by VLDL and chylomicrons, along with LDL and HDL (11, 41). ApoC-III plays a key role in the regulation of TG metabolism through the inhibition of lipoprotein lipase (LPL), hepatic receptor-mediated clearance of TG-rich lipoproteins (TRL), and promotion of VLDL production, and a direct correlation has been established between circulating levels of ApoC-III and CVD (42–44). ApoC-III is modified with O-linked glycan with multiple molecules of sialic acid at the termini (21). Three major glycoforms (or “sialoforms”) are known, referred to as ApoC-III0b, ApoC-III1, and ApoC-III2 containing 0, 1, and 2 molecules of sialic acid per molecule of protein, respectively. In plasma, the non-glycosylated form of ApoC-III (ApoC-III0a) is present at very low levels, with glycosylated forms (both non-sialylated and sialylated) representing majority of circulating ApoC-III (45). A number of studies demonstrated that the relative abundance of ApoC-III sialoforms are altered under various pathological conditions, including obesity, metabolic syndrome, diabetes, hyperlipidemia, and CVD (44–47). A correlation has been also reported between higher levels of ApoC-III1 and an atherogenic lipid profile, including increased levels of plasma total cholesterol, LDL-cholesterol, TG, and decreased levels of ApoAI (46). This report did not find any differences in the relative abundance of the three sialoforms in their ability to inhibit LPL activity, and it remains unclear how the sialylation impacts the function of ApoC-III. However, one study indicated that different ApoC-III sialoforms may influence the clearance of TRL in the liver (48). Hepatic TRL clearance is mediated by heparan sulfate proteoglycan syndecan-1, LDL receptor (LDLR), and LDLR-related protein 1 (LRP1) (49–53). The study found that LDLR and LRP1 rapidly clear TRL containing ApoC-III1, whereas ApoC-III2 is more slowly cleared by the heparan sulfate proteoglycan syndecan-1 (48). Furthermore, individuals with a loss-of-function mutation in the gene encoding polypeptide N-acetylgalactosaminyltransferase 2 (GALNT2), an enzyme involved in the glycosylation of ApoC-III, displayed sixfold increase in the levels of ApoC-III0 with a reduction in ApoC-III1, and they displayed an improved postprandial TG clearance (54).

ApoE is another glycosylated apolipoprotein that is found in various lipoproteins, including HDL, VLDL, and LDL (55). ApoE is synthesized and secreted by many cell types, including hepatocytes, smooth muscle cells, and macrophages, and it is involved in cholesterol transport and metabolism as a surface component of the lipoprotein particles (56, 57). ApoE proteins are highly O-glycosylated with terminal sialic acid residues (58–60). It is reported that desialylation of ApoE by *ex vivo* neuraminidase treatment lowers its affinity for HDL (61). This work further demonstrated that reconstituted HDL containing desialylated ApoE has lower ability to facilitate esterified cholesterol uptake in HepG2 cells and that enzymatic re-sialylation of desialylated-ApoE by sialyltransferases restores capacities to bind HDL and to take up esterified cholesterol to the levels comparable to intact ApoE (61). In addition, a potential contribution of negative charges attributed to terminal sialylation of ApoE has been implicated in the atherogenic nature of electronegative L5 LDL (62–64).

These observations collectively demonstrate the importance of sialylation in modulating lipoprotein metabolism and cardiovascular disease phenotypes in human. However, how changes in sialylation of the lipoproteins regulate the processes in the atherosclerosis and cardiovascular diseases are yet to be fully understood. Aiming to provide novel insights into the potential basis for contribution of apolipoprotein sialylation in atherosclerosis, this review focuses on the published works, in which the well-established hyperlipidemia-induced atherosclerosis mouse models were used in combination with clear gain- or loss-of-function approaches *in vivo*. More comprehensive reviews on protein or lipid glycosylation in lipid metabolism and atherosclerosis can be found elsewhere (11, 65, 66). A number of excellent reviews are also available that provide in-depth discussion of the biology of sialic acids and their binding partners and functions of the enzymes involved in sialylation processes in human health and diseases (10, 67–70).

## Mouse models of sialylation and atherosclerosis

### Administration of N-acetylneuraminic acids

A few studies demonstrated that administration of N-acetylneuraminic acid (NANA), a major form of sialic acid in mammals (71), decreases atherosclerosis in hyperlipidemic mouse models (72, 73). Guo et al. first reported (72) that NANA administration in Apoe^−/−^ mice decreased atherosclerotic plaque formation and lipid accumulation in the liver. The reduction in atherosclerosis was associated with upregulation of hepatic proteins related to RCT, such as ATP-binding cassette transporter (ABC)G1 and ABCG5 and with downregulation of inflammatory markers. More recently, Hou et al. also showed that NANA supplementation in Apoe^−/−^ mice enhances RCT, indicated by increased [^3^H]-cholesterol transfer from [^3^H]-cholesterol-loaded macrophages to the plasma, liver, and feces for excretion (73). This improvement of RCT in the latter work was associated with upregulation of ABCG1 and peroxisome-proliferator-activated receptor α (PPARα) in the liver.

### Neuraminidases

Neuraminidases (also known as sialidases) catalyze the removal of terminal sialic acids from glycoproteins, oligosaccharides, and glycolipids, and there are four mammalian subtypes of neuraminidases (NEU1–4) that show distinct, but overlapping, tissue expression, intracellular localization, and substrate specificity (74, 75). As described above, hyposialylation of lipoproteins is closely associated with atherosclerosis, and the neuraminidases are likely responsible for producing the hyposialylated forms of LDL and HDL. In addition, the enzymes may play a role in vascular inflammation in an earlier step of atherogenesis. Both NEU1 and NEU3 are expressed in human endothelial cells, and NEU1 levels negatively correlate with a capacity of endothelial cells to migrate (76). Neuraminidase-mediated removal of sialic acids from leukocyte β2‐integrin or endothelial adhesion molecule ICAM1 has been shown to enhance interaction between leukocytes and endothelial cells, contributing to proatherogenic vascular inflammation (77).

Impacts of gain- or loss-of-function manipulation of neuraminidases on cardiovascular phenotypes in mice have been reported by several groups. NEU1 is mainly expressed in lysosome and in the plasma membrane of mammalian cells (78, 79). The contribution of NEU1 in atheroprotection in mice has been first recognized as a key component of atherogenic action of elastin-derived peptide (EP) (80). EP is produced by protease degradation of the extracellular matrix of the arterial wall, and it shows multiple proatherogenic effects, including stimulation of monocyte migration, production of reactive oxygen species and oxidized LDL, and vascular smooth muscle cell proliferation (81–83). EP exerts these effects by activating the elastin receptor complex (ERC), which is composed of an elastin-binding protein, cathepsin A, and NEU1. In particular, NEU1 has been identified as the protein critically involved in signal transduction induced by ERC in various cell type (84–86). Gayral et al. (80) found that Apoe^−/−^ or Ldlr^−/−^ mice injected with EP have increased fatty streak lesions, without modifying plasma cholesterol levels. An involvement of NEU1 in atherogenesis was further investigated in NEU1-deficient mice with cathepsin A hypomorph (CathA^S190A‐Neo^), which shows a 90% reduction in NEU1 activity without developing severe sialidosis-like phenotypes associated with complete NEU1 knockout mice (87–89). Using bone marrow (BM) transplantation from CathA^S190A-Neo^ to Ldlr^−/−^-recipient mice, they showed that the mice with decreased NEU1 in hematopoietic lineage cells had decreased atheroma formation and leukocytes infiltration (80). These results suggest that specific activation of ERC by EP containing NEU1 in macrophages contributes to atherosclerosis.

To directly test whether NEU1 impacts atherosclerosis, White et al. (90) employed the mice with reduced NEU1 activity caused by the mutations in NEU1 gene (hypomorphic Neu1) (91, 92). In this study, the hypomorphic Neu1 mice on Apoe^−/−^ background (Neu1hypoApoe^−/−^) showed a reduced atherosclerotic lesion compared with the Apoe^−/−^ mice (90). The lesion in Neu1hypo;Apoe^−/−^ mice displayed fewer macrophages, T cells, and smooth muscle cells, implying attenuation of inflammation and cell recruitment within the plaque. Serum total cholesterol levels associated with VLDL and LDL were lower, and hepatic cholesterol content was decreased in Neu1hypoApoe^−/−^ mice, associated with a lower production rate of VLDL-TG, compared to Apoe^−/−^ mice. BM transplant experiments were further performed to determine the contribution of NEU1 in hematopoietic cells. Transplantation of Apoe^−/−^ BM into Neu1hypo;Apoe^−/−^ mice led to an increased atherosclerotic-lesion compared with transplantation of Neu1hypo;Apoe^−/−^ BM. The hepatic and serum lipid levels were not different between Neu1hypo;Apoe^−/−^ mice transplanted with BM from Apoe^−/−^ and Neu1hypoApoe^−/−^ donors. Furthermore, compared with that in control mice, tumor necrosis factor alpha (TNFα) treatment results in reduced leukocyte–endothelial cell interaction in Neu1hypoApoe^−/−^ mice. The rescue of the neuraminidase expression *in vivo* by adenovirus-mediated overexpression of NEU1 reduced the leukocyte–endothelial cell interaction. These data indicate NEU1 plays an important role in both leukocyte rolling and adhesion, which are important steps leading to leukocyte extravasation. Moreover, smooth muscle cell content in the aortic sinus of Neu1hypo;Apoe^−/−^ was reduced compared with that of Apoe^−/−^ mice, consistent with reports that NEU1-dependent desialylation increases smooth muscle cell proliferation (83). The reduction in macrophages in the lesion also correlates with a substantial decrease in subsets of neutrophils/granulocytes in the peripheral blood. Heimerl et al. subsequently reported that Neu1hypo mice display decreased left ventricular (LV) dysfunction after ischemia/reperfusion (I/R) injury, along with fewer pro-inflammatory macrophage infiltration (93). Wild-type (WT) mice transplanted with BM derived from Neu1hypo mice and Neu1hypo mice with WT BM both displayed better LV function after I/R compared with WT mice with WT-BM. In contrast, they found that mice with a cardiomyocyte-specific NEU1 overexpression have increased cardiomyocyte hypertrophy and reduced LV dysfunction despite a similar infarct scar size to WT mice after I/R.

More recently, Demina et al. reported that deficiency of NEU1 and NEU3 but not NEU4 attenuates atherosclerosis (94). In this study, NEU1-deficient (CathA^S190A‐Neo^), *Neu3^−/−^* or *Neu4*^−/−^ mice (87, 95, 96) were crossed with Apoe^−/−^ mice, and atherosclerosis was evaluated. Apoe^−/−^;CathA^S190A‐Neo^ and Apoe^−/−^;Neu3^−/−^ mice showed smaller atherosclerotic lesions compared to Apoe^−/−^ mice. Apoe^−/−^;Neu4^−/−^ had lesion size comparable to Apoe^−/−^ mice. In contrast to the work by White et al. (90), deficiency of NEU1 or NEU3 did not affect plasma LDL cholesterol, TG, or HDL levels. The study showed reduced numbers of macrophages in atherosclerotic lesions in Apoe^−/−^;CathA^S190A‐Neo^. Interestingly, macrophage-positive areas in the lesions were similar between Apoe^−/−^;Neu3^−/−^ and Apoe^−/−^ mice, suggesting that different mechanisms may underlie reduced plaque formation in NEU1‐ versus NEU3‐deficient mice. Parallel observations were made in mice with Ldlr^−/−^ background. The work also confirmed the previous studies that ApoB in human LDL can be desialylated *ex vivo* by NEU1 and NEU3 and that desialylated LDL is taken up by cultured macrophages. They further demonstrated that BM-derived cells in the culture from ASGR knockout mice (Asgr^−/−^) display reduced uptake of desialylated LDL compared to those from wild-type mice. *In vivo*, the injection of fluorophore-labeled desialylated LDL resulted in the incorporation of lipoproteins in liver macrophages in wild-type mice but not in Asgr^−/−^ mice. These results indicate that ASGR is likely involved in desialylated LDL taken into the macrophages, contributing to increased atherosclerosis in NEU1- or NEU3-deficient mice.

In addition to the genetic loss-of-function manipulation of neuraminidases in mice, effects of chemical inhibitors were examined in several studies. White et al. reported (90) that the administration of 2,3‐didehydro‐2‐deoxy‐N‐acetyl‐neuraminic acid (DANA), a broad spectrum neuraminidase inhibitor, in Apoe^−/−^ mice for 6 weeks resulted in the attenuation of hepatic total cholesterol and cholesteryl esters compared with that in vehicle-treated control mice. Aortic sinus lesions from the DANA-treated Apoe^−/−^ mice were reduced compared with that in control mice. They performed additional control experiments using oseltamivir (also known as Tamiflu), a specific inhibitor of influenza virus neuraminidase, which does not effectively inhibit mammalian sialidases, and they found that oseltamivir did not reduce the atherosclerotic plaque formation in male Apoe^−/−^ mice. A lack of effect by oseltaminir on atherosclerosis or thrombosis in Ldlr^−/−^ mice was also recently reported (97). Demina et al. (94) similarly reported that treatment with specific NEU1 and NEU3 inhibitor (98) or more broad NEU1 inhibitor (98, 99) reduced the size of atherosclerotic lesions in Apoe^−/−^ mice. The study found that treatment with the inhibitors did not affect the plasma levels of total cholesterol, LDL-cholesterol, HDL-cholesterol, or TG (94).

### Sialyltransferases

Glycosyltransferases are also closely involved in the key events in the early stage of atherosclerosis, including generation of functional selectin ligands and regulation of leukocyte adhesion to the endothelium and subsequent extravasation (100–102). Mammalian sialyltransferases comprise 20 glycosyltransferases that facilitate the transfer of sialic acids to the terminal glycosyl group of glycoproteins and glycolipids (69, 103). Sialic acid is attached to the glycan terminus through three different linkages, namely, α2,3, α2,6, or α2,8, which are formed by the distinct sets of sialyltransferases. As mentioned above, upregulation of plaque and plasma sialyltransferase activity has been reported in patients with atherosclerosis (28). However, whether the enzymes play any role in atherosclerosis remains largely unknown.

One of the sialyltransferases, ST3 β-galactoside α-2,3-sialyltransferase 4 (St3Gal4), catalyzes the transfer of sialic acids in the α2,3 linkage to termini of N- and O-glycans. This sialic acid modification has been implicated in von Willebrand factor (vWF) synthesis and activity (104, 105). An association of single nucleotide polymorphisms (SNPs) in the St3Gal4 gene with plasma levels of vWF was reported in multiple human cohorts after adjustment for confounders such as age, BMI, hypertension, and diabetes (106). These observations suggest a possible role of St3Gal4 in hemostasis and thrombosis. In addition, St3Gal4 is critically required for selectin function, as mice deficient in the enzyme (St3Gal4^−/−^) displayed an impaired selectin ligand function and attenuated selectin-dependent leukocyte adhesion or rolling induced by various stimuli (107–110). The interaction with selectins and their glycan ligands facilitates leukocyte tethering and rolling on the vascular endothelium, thus contributing to the early phase of atherosclerosis (5, 111). Frommhold et al. (110) reported a major role for St3Gal4-mediated sialylation of the chemokine receptor Cxcr2 in triggering leukocyte arrest on inflamed microvasculature (112). Similarly, C–C chemokine receptor type 5 (Ccr5) binding to its ligands, Ccl3 and Ccl4, was reported to be strongly dependent on a sialic-acid-carrying O-glycan in the N-terminal domain of Ccr5 (113). Doring et al. subsequently showed in Apoe^−/−^ mice that St3Gal4 deficiency reduced the size of atherosclerotic areas and numbers of macrophages in the lesion, without affecting plasma cholesterol levels (112). They further demonstrated that Ccl5-induced neutrophil and monocyte extravasation into the peritoneal cavity was reduced in St3Gal4^−/−^ mice and that St3Gal4 deficiency results in a reduced binding of Ccl5 and an abrogation of Ccl5-induced arrest on TNFα-stimulated endothelium in cell culture and *ex vivo* experiments.

ST6 β-Galactoside α-2,6-sialyltransferase 1 (St6Gal1) catalyzes the α2,6 linkage to an underlying galactose residue, and its expression and activity are closely associated with the negative regulation of the immune response (114, 115). Genome-wide association studies (GWAS) have revealed that SNPs of St6Gal1 are linked to multiple inflammatory disorders, including CVD and T2DM (116–118). Zhang et al. reported that the St6Gal1 expression in aortic endothelium is inversely related to atheroma formation in Apoe^−/−^ mice (119). In cultured EA.hy926 endothelial cell line, they further showed that siRNA knockdown of St6Gal1 promoted transendothelial migration of monocytes induced by TNFα, whereas overexpression of the enzyme had opposite effect. More recently, Holdbrooks et al. showed that BM-derived macrophages from mice with myeloid-specific St6Gal1 deletion display reduced long-term activation of nuclear factor kappa B (NF-kB) by TNFα or lipopolysaccharide (LPS) (120). Furthermore, in experiments using cultured monocytes, the study implicates TNFR1 or TLR4 to be a potential substrate of St6Gal1-mediated α2-6 sialylation related to the inflammatory regulation by monocytes/macrophages. Impacts of ST6Gal1 on atherosclerosis have not yet been reported in mice; however, Oswald et al. reported (121, 122) that mice with hepatocyte-specific St6Gal1 deletion develop spontaneous hepatic steatosis after 52 weeks on high-fat diet, indicated by the accumulation of fat droplets, inflammatory cytokine production, and presence of pro-inflammatory macrophages in the liver.

## Discussion

The loss- and gain-of-function mouse models of sialylation machinery so far have provided a valuable tool to understand the molecular mechanism by which protein sialylation modulates atherosclerosis progression. These works highlight the significant contributions by the enzymes and substrates related to protein sialylation in diverse sets of cell types, including leukocytes, macrophages, endothelial cells, immune cells, and hepatocytes. The loss-of-function studies for neuraminidases demonstrate a major atheroprotective role of lipoprotein sialylation. In contrast, the studies in mice with St3Gal4 deficiency implicate that sialylation of selectin ligands and chemokine receptors likely plays an atherogenic role. These opposing functions of protein sialylation depend upon the target substrate molecules and the locations where the modification is regulated. Recent studies in mice using BM transplantation or monocyte-specific Cre-LoxP system have uncovered the key function of neuraminidases and sialyltransferases in monocytes and macrophages (80, 90, 94, 120, 123). More studies are needed to address the importance of protein sialylation using mice with additional tissue- and cell-type-specific targeting of the machinery, including endothelial cells and vascular smooth muscle cells. Similarly, in addition to the lipoproteins and the selectin ligands, a number of plasma proteins and the lipoprotein receptors are sialylated, and modulation of the sialylation status of these proteins likely play a role in atherogenesis. For example, a recent study found that sialylation of the scavenger receptor CD36 is modulated by NEU1 containing ERC complex in macrophages (124). CD36 is a scavenger receptor expressed on the surface of a wide range of cells, including macrophages, platelets, and microvascular endothelial cells, and CD36 deficiency has profound atheroprotective effects in mice (125, 126); however, whether the sialylation of CD36 contributes to atherogenic effect of ERC is yet to be proven. Another study in humans investigated an association of glycosylation and sialylation traits of plasma immunoglobulin (IgG) with subclinical atherosclerosis (127). The work identified specific traits related to IgG sialylation that are negatively correlated with cardiovascular disease risk, circulating levels of VLDL and TG, and presence of carotid plaque. Increased levels of IgG with low sialylation and glycosylation have been observed in patients with inflammatory diseases such as rheumatoid arthritis and Crohn’s disease (128, 129). In mouse models, increase in IgG sialylation *in vivo via* provision of the sialic acid precursor or engineered transferases resulted in attenuation of the inflammation-associated disease outcomes (128, 130). Future investigations are warranted to determine whether hyposialylation of IgG plays a similar pathogenic role in atherosclerosis in mouse models or in human.

Finally, close associations between genetic polymorphisms of enzymes and receptors related to sialylation and cardiovascular risks have emerged. For example, in a genetic study of Icelanders, researchers discovered a rare noncoding 12-bp deletion (del12) in the Asgr1 gene that generates a premature stop (131). They found that this variant form of Asgr1 is strongly associated with a decrease in plasma levels of non-HDL cholesterol and TG and with reduced risk for CVD. Mirroring the human phenotype, a recent study showed that Asgr1^−/−^ mice exhibit lower non-HDL-cholesterol and TG caused by decreased secretion and increased uptake of VLDL/LDL (132). Similar approaches combining human genetics with preclinical mouse models will further advance the understanding of how protein sialylation impacts atherosclerosis and CVD. However, a recent report by Kawanishi et al. adds more complexity in application of the findings in non-human models to humans (133). The work demonstrated that a loss of cytidine monophosphate-N-acetylneuraminic acid (Neu5Ac) hydroxylase (CMAH) contributes to the development of atherosclerosis. CMAH catalyzes the generation of N-glycolylneuraminic acid (Neu5Gc) from its precursor Neu5Ac in majority of mammals including mice, but humans lack CMAH due to pseudogenization of the gene, resulting in a species-specific Neu5Gc deficiency in humans (134, 135). Mice with Cmah deficiency, mimicking human-like Cmah pseudogenization, on Ldlr^−/−^ background developed increased atherosclerosis compared to single Ldlr^−/−^ mice (133, 136). The use of the humanized mouse model needs to be taken into consideration for future preclinical studies.

Although an alteration of sialylation in plasma lipoproteins has long been associated with atherosclerosis and CVD, the field of sialylation in atherosclerosis is still in its infancy. Emerging works in mice discussed in this review have established that sialylation is a key mechanism that influences atherosclerosis. Future studies are urgently needed to fill in the major knowledge gaps—paucity of loss-of-function mouse models for additional enzymes and transporters and limited information regarding the sialylated proteins involved in atherosclerosis.

## Author contributions

All three authors contributed to reviewing of the literature and writing of the manuscript. All authors contributed to the article and approved the submitted version.

## Funding

The work is funded by NIH R01 (5R01DK110127, 5R01DK110127-S1, CM).

## Conflict of interest

The authors declare that the research was conducted in the absence of any commercial or financial relationships that could be construed as a potential conflict of interest.

## Publisher’s Note

All claims expressed in this article are solely those of the authors and do not necessarily represent those of their affiliated organizations, or those of the publisher, the editors and the reviewers. Any product that may be evaluated in this article, or claim that may be made by its manufacturer, is not guaranteed or endorsed by the publisher.
